# Management strategies for sport-related traumatic dental injuries: a systematic review based on case reports

**DOI:** 10.1186/s13102-025-01188-1

**Published:** 2025-07-19

**Authors:** Alice Corrêa Silva-Sousa, Jéssica Oliveira-Aguiar, Francisco Wanderley Garcia Paula-Silva, Manoel Damião Sousa-Neto, Amanda Pelegrin Candemil

**Affiliations:** 1 Department of Pediatric Dentistry, School of Dentistry of Ribeirão Preto, University of São Paulo (USP), Ribeirão Preto, SP Brazil; 2 Department of Restorative Dentistry, School of Dentistry of Ribeirão Preto, University of São Paulo (USP), Ribeirão Preto, SP Brazil

**Keywords:** Athletic Injuries, Dental Trauma, Injury Management, Sports, Sports Dentistry, Tooth Injuries

## Abstract

**Background:**

Recent advancements in sports dentistry have emphasized the critical need for effective management and outcomes assessment of sports related dental injuries. The aim of this systematic review was to compile and analyze case reports and to explore various strategies in treating sports-related dental trauma and thei r outcomes.

**Methods:**

A comprehensive search of PubMed, Scopus, and Web of Science databases, covering the period from 2005 to 2024, was conducted to identify relevant studies focusing on dental injuries among athletes among case reports on a wide range of injuries such as avulsions, fractures, and soft tissue trauma, during sports activities. Two independent researchers conducted the screening and bias assessments using predefined criteria.

**Results:**

The review includes 8 studies, revealing diverse treatment managements and outcome assessment across different sports activities and injury types. Sports-related dental trauma presents a significant challenge in clinical practice, impacting athletes’ oral health and performance. The review underscores the importance of early intervention, adherence to established guidelines, and comprehensive management protocols to optimize treatment outcomes and prevent long-term complications suggesting an evolving role of dentistry in sports medicine and heightened awareness among athletes, coaches, and healthcare providers.

**Conclusions:**

In conclusion, the management of sports dental trauma is highly heterogeneous among different or the same type of sport, highlighting the need for better coach education on IADT guidelines and preventive strategies such as mouthguard use. Future research should address barriers to guideline implementation and evaluate preventive interventions.

**Supplementary Information:**

The online version contains supplementary material available at 10.1186/s13102-025-01188-1.

## Background

Dental trauma is a common occurrence, especially among children and adolescents, and can result from accidents, falls, or sports activities. The most frequent dental injuries include crown fractures, root fractures, luxations, and avulsions [1]. Studies indicate that one-third of preschool children experience some form of dental trauma during childhood [2–8], with a higher prevalence among adolescents involved in contact sports [1]. The prognosis of these injuries varies depending on their severity and type, as well as the adequacy of timely instituted treatment [9]. Simple crown fractures generally have a good prognosis if treated quickly, while luxations and avulsions can have complications like pulp necrosis and root resorption, compromising the longevity of the affected tooth [9]. Prevention, early diagnosis, and appropriate treatment are crucial to improving long-term outcomes and minimizing the impact of these injuries on the oral health and the quality of life of patients [10, 11].

Engaging in sports activities is essential for promoting health and well-being, but poses risks, including those related to dental trauma and are a challenge for dental surgeons [12]. Studies indicate that the incidence of dental trauma is high in contact sports like football, basketball, and martial arts, where the likelihood of direct impact on the facial area is greater [13–16]. Combat sports have a high prevalence (around 80%) of facial injuries [17].

Sports dentistry aims to prevent, diagnose, and treat oral and maxillofacial and dental injuries in athletes, during sports activities [18]. The field is responsible for disseminating information about dental trauma and identifying problems that affect athletes’ performance, such as mouth breathing, unsatisfactory occlusion, and the use of medications that do not contain certain prohibited substances commonly found in analgesic formulations [19]. Athletes often have worse oral health compared to the general population, leading to acute and chronic problems that can compromise performance, and preventive measures must be taken to avoid the possibility of losing years of preparation for competitions due to acute dental problems [19]. The need for an interdisciplinary approach involving dentists, sports physicians, physiotherapists, and other healthcare professionals is of paramount importance [12]. Integrating this knowledge in the real world setting is fundamental for the implementation of appropriate preventive measures and the effective management of dental trauma [12, 19].

This review was guided by the research question: How is the clinical management of sports-related dental trauma described in in-vivo studies? Accordingly, the study aimed to compile and analyze case reports between 2005 and 2024 and to explore various strategies in treating sports-related dental trauma and their outcomes. Therefore, sports dentistry enhances the safety and performance of athletes and contributes to their quality of life after their sports careers are over by preventing long term sequalae arising from dental trauma [11]. This expanding field offers a unique opportunity for research and development of new technologies and treatment protocols, reflecting a continuous commitment to the holistic health of athletes [19]. In this context, it becomes relevant to review the case reports in the literature regarding the management of sport-related trauma cases.

## Methods

### Protocol and study design

This systematic review followed the Preferred Reporting Items for Systematic Reviews and Meta-Analysis (PRISMA) [20] checklist (Supplementary Table 1), and the protocol was registered in the International Prospective Register of Systematic Reviews (PROSPERO-2024 CRD42024559291). Based on this protocol, the review explored various aspects of managing sports dental trauma.

This review searched for all relevant *in-vivo* studies concerning the management strategies for Sport-Related Traumatic Dental Injuries, covering the period from 2005 to 2024. To assess all the articles of the theme, a guiding question and an eligibility strategy were used in the review process. The guiding question of the study was the following: How was the clinical management of sports dental trauma among *in-vivo* studies? The PICO (Patient, Intervention, Comparison, and Outcome) strategy was used to define the key elements of the guiding question. The study population (P) consisted of patients with sports dental trauma, the intervention (I) involved studies that performed management of sports dental trauma in case reports, the comparison (C) was not considered. The outcome (O) evaluated the management of sports dental trauma (luxation, fracture, extrusion, intrusion, movement, avulsion, dislocation) of different case reports.

### Literature search strategy

The search strategy, as described in Table 1, included only studies indexed in the following electronic databases: PubMed, Scopus, and Web of Science. A search was also conducted in the gray literature. The studies were limited to those available in English. The search was performed using terms from Medical Subject Headings, or Text Word combined with the Boolean connectors “OR” and “AND” to encompass the key elements from the guiding question.

**Table 1:** Search strategy as a function of the database

PubMed: 21 document results
((("Traumatic dental injuries"[Mesh] OR"Dental trauma"[Title/Abstract] OR"Luxation injuries"[Title/Abstract] OR"Tooth Luxation"[Title/Abstract] OR"Dental injuries"[Title/Abstract] OR"Injuries, Teeth"[Title/Abstract] OR"Injuries, Tooth"[Title/Abstract] OR"Teeth Injuries"[Title/Abstract] OR"Tooth Injuries"[Title/Abstract] OR"Tooth Fractures"[Title/Abstract] OR"Crown fractures"[Title/Abstract] OR"Enamel fracture"[Title/Abstract] OR"Enamel/dentin fracture"[Title/Abstract] OR"Crown fracture"[Title/Abstract] OR"Crown/root fracture"[Title/Abstract] OR"Root fracture"[Title/Abstract] OR"Alveolar fracture"[Title/Abstract] OR"Extrusion"[Title/Abstract] OR"Lateral luxation"[Title/Abstract] OR"Tooth Intrusion"[Title/Abstract] OR"Tooth Movement, Minor"[Title/Abstract] OR"Avulsed Tooth"[Title/Abstract] OR"Dislocation, Tooth"[Title/Abstract] OR"Tooth Avulsion"[Title/Abstract] OR"Facial Injury"[Title/Abstract]) AND ("Diagnosis"[Mesh] OR"Dental trauma Diagnosis"[Title/Abstract] OR"Dental trauma management"[Title/Abstract] OR"Therapeutic decision"[Title/Abstract] OR"Decision making"[Title/Abstract] OR"Clinical decision"[Title/Abstract] OR"Clinical decision making in dental trauma"[Title/Abstract])) AND ("Sport"[Mesh] OR"Sports"[Title/Abstract] OR"Sport activity"[Title/Abstract] OR"Sport-Related"[Title/Abstract] OR"Basketball"[Title/Abstract] OR"Soccer"[Title/Abstract] OR"Athletic Training"[Title/Abstract] OR"Players"[Title/Abstract] OR"Water Polo"[Title/Abstract])) AND ("Tooth"[Mesh] OR"Dental"[Title/Abstract])
**Scopus: 34 document results**
(TITLE-ABS-KEY (traumatic AND dental) OR TITLE-ABS-KEY (dental AND trauma) OR TITLE-ABS-KEY (tooth AND injuries) AND TITLE-ABS-KEY (dental AND trauma AND diagnosis) AND TITLE-ABS-KEY (sport) OR TITLE-ABS-KEY (sport AND related) OR TITLE-ABS-KEY (sports) AND TITLE-ABS-KEY (dental) OR TITLE-ABS-KEY (tooth))
**Web of Science: 304 document results**
Traumatic dental injuries (All Fields) and Sport (All Fields) and Dental (All Fields)

### Study selection and screening process

Two researchers (A.C.S.S and A.P.C) independently screened titles and abstracts of the identified studies and selected those that met the eligibility criteria described below. Disagreements were resolved through consensus. The full text of the selected studies was then assessed to determine if they aligned with the present guiding question. Both A.C.S.S and A.P.C. independently reviewed these studies to decide which ones to include in this systematic review, with any disagreements resolved through consensus. Additionally, one researcher (J.O.A) assessed the selected studies to identify additional ones that could address the guiding question. Microsoft Excel (Microsoft Corporation, Redmond, WA) was used to register the decisions.

### Inclusion and exclusion criteria

The inclusion criteria encompassed all studies evaluating the clinical management of sports related dental trauma such as luxation, fracture, extrusion, intrusion, movement, avulsion, and dislocation of the tooth, in both adult and pediatric populations. The selected studies examined *in-vivo* case reports and were indexed in the search.

Exclusion criteria were studies using animals, *ex-vivo* studies, review studies, systematic reviews, editorials, and book chapters. Studies unavailable for full-text assessment were also excluded.

### Data extraction

The data extraction included general information that identified the study (author and year), Patient information (gender and age), study objective, first aid, type of sport, use of prevention, diagnostic tests, management, and follow-up.

### Risk of bias assessment

The risk of bias of the included articles was assessed by two evaluators using the Joanna Briggs Institute (JBI) critical appraisal checklist for case reports which comprises 8 items evaluating aspects such as patient demographic characteristics, history, clinical condition, diagnostic tests, intervention details, clinical condition post-intervention, adverse events and takeaway lessons. Each item was scored as “Yes,” “No,” “Unclear,” or “Not applicable.” [21–23]. The final score of each article was calculated based on the percentage of negative answers and classified as having a ‘high’ risk of bias [score > 70%], ‘moderate’ risk of bias [score from 50%−69%], and ‘low’ risk of bias [score ≤ 49%]. Discrepancies between evaluators were resolved by discussion or consultation with a third reviewer to ensure consistency.

## Results

### Selected studies

**Fig. 1:**
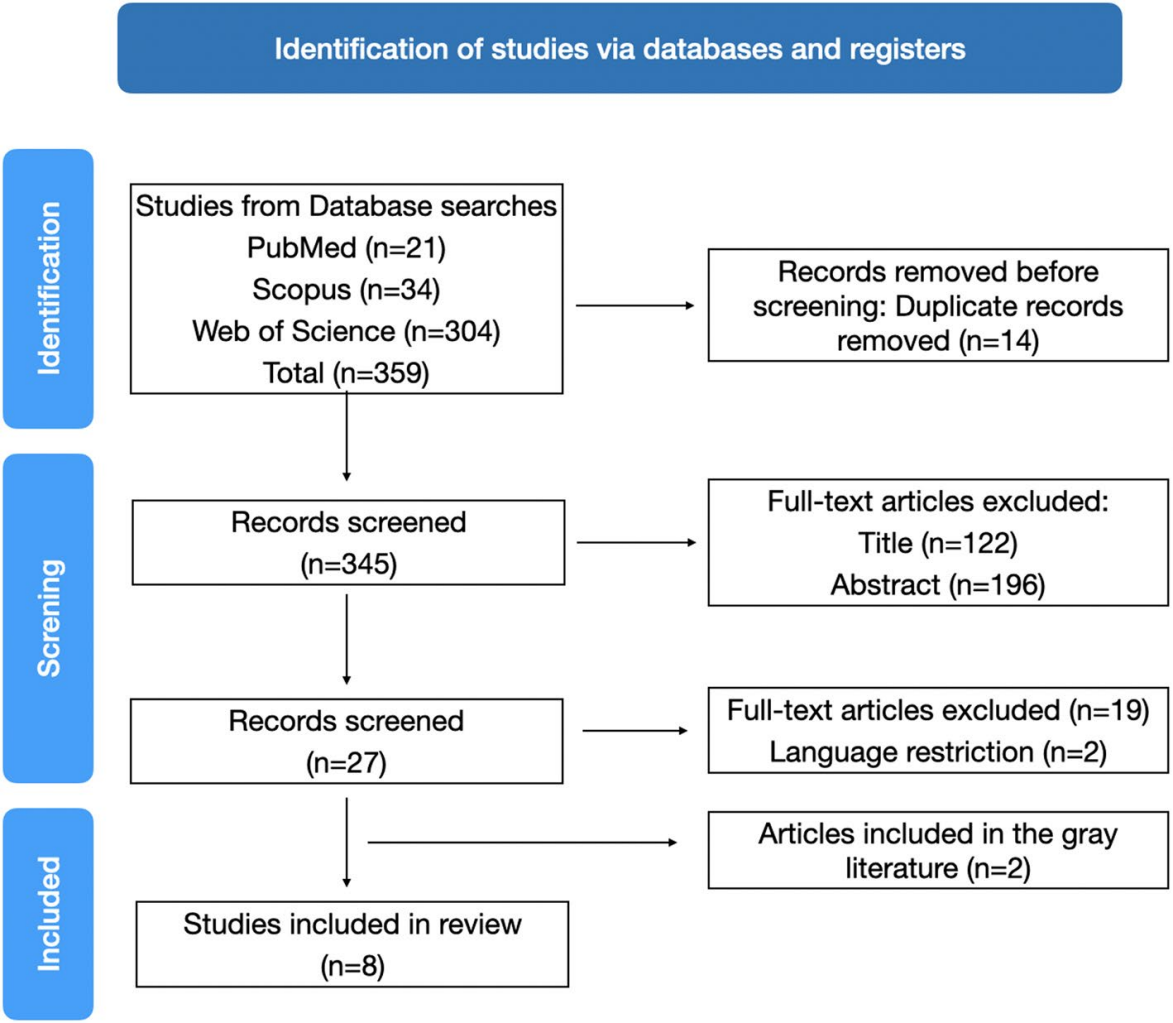
A Flow Diagram of the study selection following the Preferred Reporting Items for Systematic Reviews and Meta-Analysis statements (PRISMA)

A total of 359 studies were preselected; 14 were excluded due to duplication. The titles and abstracts of the 345 remaining studies were carefully reviewed, leading to the exclusion of 318 studies. Then, after assessment of the full text, 19 studies were excluded due to not being aligned with the present guiding question, 19 studies were excluded and 2 because of not being available in English language. Two studies were included through the search conducted in the gray literature. Therefore, 8 studies were included in this systematic review (Fig. 1). The level of agreement between the reviewers, calculated using Kappa’s coefficient, was considered excellent (0.99).

The search strategy aimed to include all types of studies involving human patients, including observational, cohort, and prospective studies. However, after applying the predefined inclusion criteria, only case reports met the eligibility requirements. This reflects the current state of the literature, highlighting the need for further high-quality research on the management of sports-related dental trauma.

### Characteristics of the studies

The publication years of the selected studies ranged from 2005–2022, with patients of an age range of 9–36 years. Concerning the sports event related to the trauma, 37.5% (*n* = 3) were basketball, 25% (*n* = 2) soccer, 12.5% (*n* = 1) golf, 12.5% (*n* = 1) volleyball, and 12.5% (*n* = 1) bicycle. The studies evaluated a variety of injuries including dental fractures, avulsion, intrusion, and bone fracture. The percentage of trauma to the incisors was 75%, 12,5% dental (not specified dental group) and 12.5% bone trauma. Successful outcomes occurred in 62.5% of the cases. In all cases the athletes were not using mouth guards or other forms of prevention at the time of trauma. The investigations prior to initial management were periapical radiography (62.5%), cone-beam CT (25%), and ultrasound (12.5%). The follow-ups included periapical radiography (62.5%), cone-beam CT (12.5%), and ultrasound (12,5%), the others studied (25%) did not use complementary examinations for follow-up. The summary of this extraction is presented in Tables 2, 3 and 4.

**Table 2:** Data extraction from the included studies related to trauma and the first aid provided

Author and year	Demographics	Sport	Type and region of Trauma	First aid			Did any prevention method was used?
				Time since Trauma	**History**	**Clinical Management**	
Mihalik et al. 2005 [31]	Male 17 years	Soccer	Fracture—Maxila	Immediately after	Evaluated in an emergency room, revealed evidence of a complicated upper lip laceration, multiple facial fractures, and a closed head injury	None	No
Karp et al., 2006 [27]	Male 9 years	Golf	Avulsion—Left central incisor	Immediately after	First, the tooth was placed in water for 10 min, followed by 10 min in cold milk before being returned to its socket	The tooth was replaced in its socket by the family	No
Santos et al., 2006 [30]	Male 36 years	Basketball	Fracture—Malar bone	x	Evaluated by a maxillofacial surgeon	x	No
Stojanac et al.; 2016 [24]	Male 21 years	Basketball	Fracture—Maxillary right lateral and both central incisors	One day after	Evaluated by a maxillofacial surgeon which referred to the Department of Restorative Dentistry and Endodontics	Debridement of the wounds, and prescription of antibiotics	No
Dello Diago et al., 2020 [29]	Male 13 years	Basketball	Intrusive dislocation—Maxillary right central and lateral incisor	x	During an emergency visit the patient was evaluated	Teeth had been repositioned, treated endodontically and splinted with a multi-bracket orthodontic	No
Musu et al., 2021 [25]	Female 25 years	Volleyball	Fracture—Enamel-dentinal of maxillary right and left central incisors (history of concussion 6 years before)	6 years after	The patient manifested a periodical discharge of pus in the trauma area	Antibiotic therapy and self-performed drainage	No
Moraes et al., 2021 [26]	Male 36 years	Soccer	Fracture—Maxillary left central incisor	Two hours after the trauma	The immediate treatment was performed in a private clinic	The tooth was manually repositioned, and splinting with light-curing resin	No
Chikkanarasaiah et al., 2022 [28]	Male 15 years	Bicycle	Avulsion—Maxillary right lateral and both central incisors	1 and a half months after	Patient’s mother stored the teeth in saline a solution	None	No

**Table 3:** Data extraction from the included studies related to imaging examinations and clinical management following image-based diagnosis

Author and year	Sport	Periapical Radiography		CBCT		Ultrasound		Management After Image Diagnosis	
		**Time since trauma**	**Diagnosis**	**Time since trauma**	**Diagnosis**	**Time since trauma**	**Diagnosis**	**Time since trauma**	**Therapy**
Mihalik et al. 2005 [31]	Soccer	x	x	On the day	Fractures of the anterior, posterior,lateral and medial walls of the right maxillary sinus and of the floor of the right orbit	x	x	3 weeks after	Orthodontic therapy, gingival surgery and nonsurgical endodontic treatments
Karp et al., 2006 [27]	Golf	On the day	Immature incisors with open apices	x	x	x	x	On the day	The left central incisor was repositioned and a 50-lb monofilament fishing line splint was stabilized with composite resin
Stojanac et al.; 2016 [24]	Basketball	One the day	Horizontal fractures on the maxillary right lateral incisor and both maxillary central incisors, labially in the cervical third and extending subgingivally on the palate, with exposed pulp tissue	x	x	x	x	One the day	The fragments were temporarily repositioned and splinted using a composite-resin splint. Root Canal Treatment, followed by definitive repositioning of fragments two days later after raising a full-thickness gingival flap. The teeth were restored with composite resin and the patient was instructed on oral hygiene, nutrition, implementation of precautions, and the necessity of using a mouthguard during sports activity
Musu et al., 2021 [25]	Volleyball	6 years after	Unsuccessful root canal treatment and periapical radiolucency on right lateral incisor. A subsequent radiograph, with a gutta-percha cone introduced into the tract opening, traced the route of the drainage and identified the periapical lesion on the right lateral incisor as the source of this condition	x	x	6 years after	Echogenic, solid lesion with a poorly defined hyperechoic bone outline and internal blood vessels, suggestive of an apical granuloma. Ecographic representation of the sinus tract, which interrupted the cortical plate and exhibited a dishomogeneous, hypoechoic pathway surrounded by echogenic and reinforced boundaries on right lateral incisor	6 years after	Root canal retreatment of right lateral incisor was peformed. Periapical radiography showed insatisfactory quality of apical instrumentation due to difficulties in removing the previous fill and reaching the full working length. It was planned to wait three months before a surgical endodontic treatment
Dello Diago et al., 2020 [29]	Basketball	14 days after	Failure of the reimplantation	x	x	x	x	14 days after	Splint was removed, the mobility of both teeth (degree 2 on the Miller scale) was evaluated and the maxillary right central and lateral incisor were extracted. Orthodontic treatment was performed on both arches to maintain spaces and harmonize the occlusion. A removable partial denture was delivered to restore the edentulous areas that were surgically exposed, and the surgical guide was positioned
Chikkanarasaiah et al., 2022 [28]	Bicycle	x	x	x	x	x	x	1 and a half months after	Biological removable functional space maintainer was planned using patient’s natural teeth. Postoperative instructions were given for the patient to clean it before sleeping and store the space maintainer in water at night. The patient was observed until his growth is complete
Moraes et al., 2021 [26]	Soccer	Three days after	Oblique horizontal radicular fracture in the cervical third	x	x	x	x	Three days after	Splint maintenance for three months associated with custom-made mouthguards to guarantee the athlete’s safe return to sports practice
Santos et al., 2006 [30]	Basketball	x	x	On the day of trauma	Left malar bone had been fractured into three points (two in the anterior and one in the lateral region)	x	x	On the day	No therapy was necessary in the face of the diagnosis

**Table 4:** Data extraction from the included studies concerning patient follow-up after initial treatment

Author and year	Type of Trauma	First follow-up					Second follow-up				
		**Time**	**Periapical**	**CBCT**	**US**	**Therapy**	**Time**	**Periapical**	**CB CT**	**US**	**Therapy**
Mihalik et al. 2005 [31]	Fracture	x	x	x	x	x	x	x	x	x	x
Karp et al., 2006 [27]	Avulsion	12 days after	The left central incisor was stable with good gingival healing and physiologic mobility. The maxillary incisors revealed a normal response to percussion testing, and radiographically displayed no evidence of pathology	x	x	The splint was removed, and the patient was reappointed for an additional follow-up visit in 1 month	47 days after	External inflammatory root resorption encompassing the root structure of the middle to cervical thirds of the left central incisor	x	x	Apexification process
Stojanac et al.; 2016 [24]	Fracture	7 months after	Fractured tooth (previously restored—again occurred during a basketball game)	x	x	Indicated for extraction	x	x	x	x	x
Musu et al., 2021 [25]	Fracture	6 years and 3 months after	x	Large bone loss of the periapical areas of right central and lateral incisors	Sinus tract pathway was still present and exhibiting a high degree of inflammation	Surgical endodontic treatment, removing the periapical lesion, resecting 3 mm of the root-end, and retrograde obturate was performed	6 years and 4 months after	x	x	x	Persistence of the active sinus tract. A cavity test was performed on the right central incisor, which was then diagnosed with pulpal necrosis and treated endodontically
Dello Diago et al., 2020 [29]	Intrusive dislocation	2 months after	Control	x	x	x	4 months after	x	x	x	Mini-implants were placed in the maxillary right central and lateral incisor. After 14 days, temporary resin crowns were placed, and orthodontic treatment was completed
Chikkanarasaiah et al., 2022 [28]	Avulsion	x	x	x	x	x	x	x	x	x	x
Moraes et al., 2021 [26]	Fracture	1 month	Normal and healthy appearance of the left central incisor	x	x	x	3 months	Normal and healthy appearance of the left central incisor	x	x	x
Santos et al., 2006 [30]	Fracture	3 month after	No damage to the affected teeth	x	x	x	1 year after	Absence of any damage to the teeth	x	x	The athlete become aware of the importance of wearing a mouthguard

Author and year	Third follow-up					Further follow-up				
	**Time**	**Periapical**	**CB CT**	**US**	**Therapy**	**Time**	**Periapical**	**CB CT**	**US**	**Therapy**
Mihalik et al. 2005 [31]	x	x	x	x	x	x	x	x	x	x
Karp et al., 2006 [27]	75 days after	Left central incisor revealed further external resorption of the middle to cervical root structure that appeared to encroach upon the root canal space	x	x	x	1. 166 days after 2. 422 days after 3. 865 days after	1. Cessation of the resorptive process. 3. The left maxillary central incisor with normal mobility and no evidence of replacement resorption or pathology	x	x	1. Apexification process; 2. Endodontic treatment using MTA
Stojanac et al.; 2016 [24]	x	x	x	x	x	x	x	x	x	x
Musu et al., 2021 [25]	6 years, 4 months and one week after	x	x	Disappearance of the bony tract and reduction of the inflammatory signal	x	6 years and 5 months to 9 years after	Asymptomatic and healing of the lesions	x	x	x
Dello Diago et al., 2020 [29]	9 months after	Good healing of soft tissues without infection	x	x	Temporary crowns were replaced with the final crowns. A multilayer individual EVA mouthguard was delivered to be worn during sports activities	13 years after	Implants were stable and bone volumes were maintained	x	x	x
Chikkanarasaiah et al., 2022 [28]	x	x	x	x	x	x	x	x	x	x
Moraes et al., 2021 [26]	1 year	Normal and healthy appearance of the left central incisor	x	x	x	2 years	Normal and healthy appearance of the left central incisor	x	x	x
Santos et al., 2006 [30]	x	x	x	x	x	x	x	x	x	x

### Trauma managements

#### Dental fractures

Stojanac et al. [24], Musu et al. [25], and Moraes et al. [26] provide detailed accounts of dental fractures in sports-related incidents, illustrating the varied approaches to treatment and outcomes. The three cases highlight the differences on management and prognosis of sports-related dental fractures (Table 1). Stojanac et al. [24] demonstrated the challenges of managing severe horizontal fractures with immediate and follow-up interventions (Table 2), ultimately leading to tooth loss despite initial successful treatments. Musu et al. [25] showcased the complications arising from delayed post-trauma management and previous concussions, emphasizing the importance of thorough and ongoing treatment to prevent chronic issues. In contrast, Moraes et al. [26] illustrated a successful immediate intervention and long-term management approach, resulting in the preservation and health of the fractured tooth.

Stojanac et al. [24] described the case report of a basketball player who suffered horizontal fractures of teeth. The fractures extended labially in the cervical third and subgingivally on the palate, exposing pulp tissue. One day after the trauma, a maxillofacial surgeon performed wound debridement, ruled out facial bone fractures, and prescribed antibiotics. The teeth fragments were temporarily repositioned and splinted with a composite-resin splint. Root canal treatment followed, with definitive fragment repositioning after raising a full-thickness gingival flap. The teeth were restored with composite resin. The patient returned seven months later with re-fractured teeth from another basketball game, necessitating the extraction of two previously reconstructed teeth.

In another context, Musu et al. [25] reported a case report of a volleyball player who had a history of concussion six years prior and experienced enamel-dentin fractures. Six years later, she experienced periodic purulent discharge in the same area, managed with antibiotics and self-drainage. Radiographs revealed an unsuccessful root canal treatment and periapical radiolucency, with a subsequent fistulography radiograph. An echographic evaluation suggested an apical granuloma. Surgical endodontic treatment was performed, and a three-month follow-up showed large bone loss in the periapical areas. Despite ongoing inflammation detected by ultrasound, the patient remained asymptomatic for three years with radiographic healing of the lesions.

In contrast, Moraes et al. [26] showed an oblique horizontal radicular fracture in the cervical third during an high-level soccer game. Two hours post-trauma, the tooth was manually repositioned and splinted with light-curing resin. Splint maintenance was planned for three months, alongside custom-made mouthguards to ensure a safe return to sports. Regular follow-ups at one month, three months, one year, and two years showed the tooth remained healthy and stable, demonstrating successful long-term outcomes.

### Dental avulsio

Karp et al. [27] and Chikkanarasaiah et al. [28] described two distinct cases of dental trauma involving avulsion, both highlighting different approaches and outcomes in the management of such injuries (Table 1 and 2). Karp et al. [27] described a dental avulsion case attributed to an injury during a golf game. Immediately after the injury, the tooth was stored in water for 10 min and then in cold milk for another 10 min before being reinserted into the socket by the family. The avulsed tooth had immature apices. Upon emergency treatment, the left central incisor was repositioned and splinted with a 50-lb monofilament fishing line stabilized by composite resin. 47 days post-injury, external inflammatory root resorption was noted, and the apexification process was initiated 75 days post-injury due to further external resorption. By 422 days, endodontic treatment using MTA was completed, and 865 days post-injury, the tooth exhibited normal mobility with no clinical or radiographic evidence of replacement resorption or pathology.

In another context, a trauma after a bicycle accident was reported by Chikkanarasaiah et al. [28]. The patient's mother stored the teeth in a saline solution. However, professional dental care was not sought until one and a half months after the trauma, which made reimplantation attempts not possible. Instead, a biological removable functional space maintainer was planned using the patient’s natural teeth.

### Dental intrusion

Dello Diago et al. [29] reported a traumatic intrusive dislocation case report related to a basketball game. During an emergency visit, the teeth were repositioned, treated endodontically, and stabilized with a multi-bracket orthodontic appliance. Fourteen days post-trauma, after the splint removal, the professional diagnosed unsuccessful reimplantation. Then, the teeth were extracted due to the verified mobility of degree 2 (Miller scale). As a way to maintain the space and occlusal harmony of the extracted teeth area, orthodontic treatment was initiated with the association of a removable partial denture to restore the edentulous area. Then, two mini-implants were placed in the regions of the maxillary right central and lateral incisors and temporary resin crowns were fitted, completing the orthodontic treatment. Finally, the follow-up showed good soft tissue healing, stable implants and well-maintained bone volume (Tables 1 and 2).

### Bone fracture

Santos et al. [30] and Mihalik et al. [31] describe cases of maxillofacial fractures resulting from sports-related injuries, providing insights into the management and outcomes of such traumas. The two cases highlight the varied nature of maxillofacial fractures and the importance of immediate and appropriate management (Tables 1 and 2). Santos et al. [30] emphasize the significance of preventive measures, such as wearing a mouthguard, to mitigate the risk of fractures. Despite the lack of first aid, the patient did not experience any dental damage, as confirmed by long-term radiographic evaluations. In contrast, Mihalik et al. [31] illustrate a more severe case involving multiple facial fractures and a closed head injury. Immediate evaluation and subsequent orthodontic and surgical treatments were necessary to address the complexity of the injuries. These cases underscore the necessity of prompt medical attention and tailored treatment plans to manage sports-related maxillofacial fractures effectively.

Santos et al. [30] described the case of a patient who suffered a fracture of the malar bone during a basketball game. The injury resulted in the malar bone fracturing at three points: two in the anterior region and one in the lateral region. No first aid was administered at the time of the injury. Radiographic evaluations conducted three months post-trauma showed no damage to the affected teeth. One year later, further radiographs and sensitivity tests confirmed the absence of any dental damage.

A case during a soccer game was described by Mihalik et al. [31] where the patient sustained a maxillary fracture. The injury was immediately evaluated in an emergency room, revealing a complicated upper lip laceration, multiple facial fractures, and a closed head injury. No first aid was performed at the scene. Orthodontic therapy began three weeks post-injury to align and level the teeth, followed by gingival surgery and nonsurgical endodontic treatments.

### Risk of bias

**Fig. 2:**
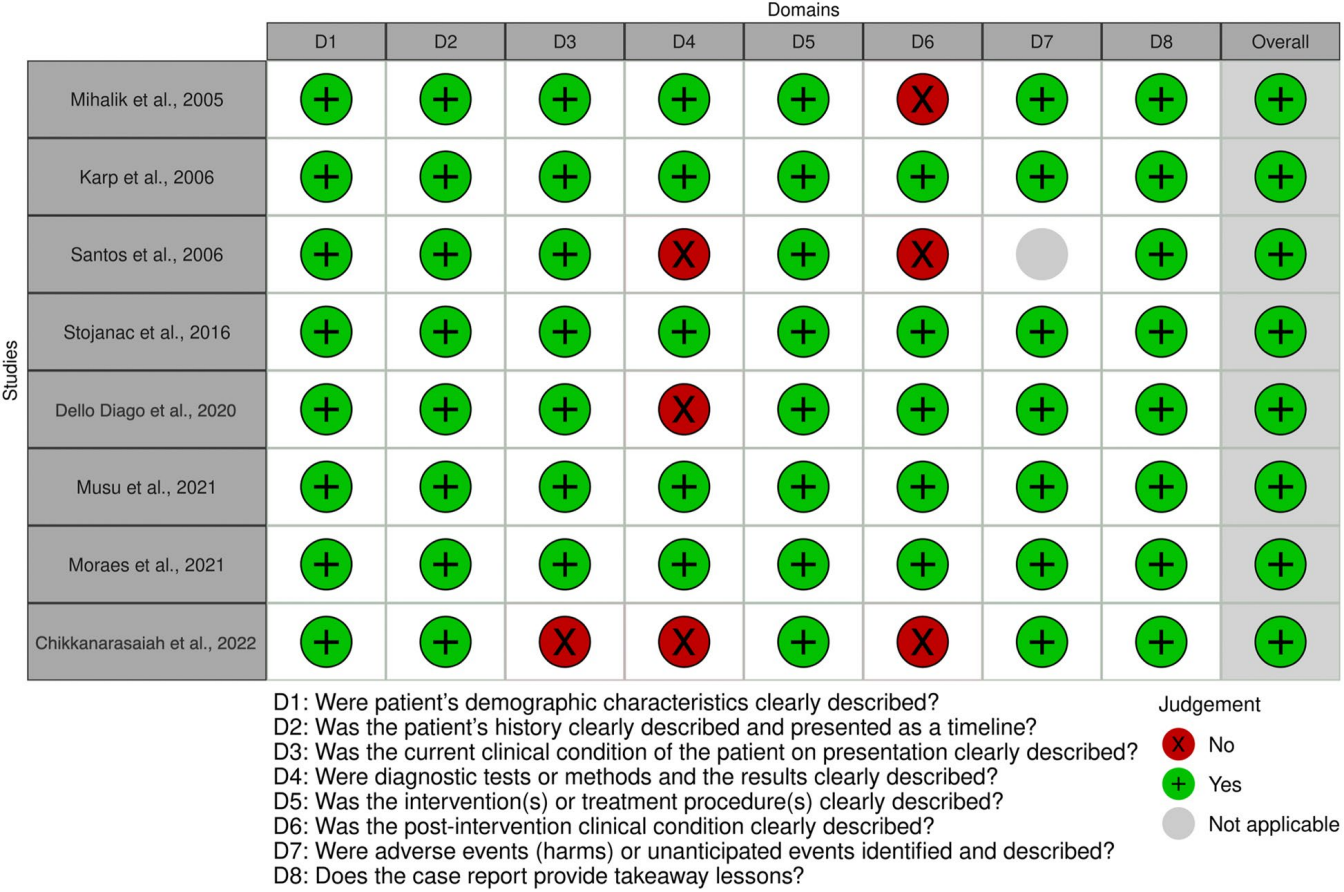
Risk of bias chart results, encompassing the assessment of 8 key domains, as well as the overall classification of the sample review. The summary plot shows the distribution of classifications across these domains

Figure 2 displays the risk of bias in the included studies. Overall, the methods used were homogeneous and showed a low risk of bias.

## Discussion

This systematic review compiled various reports on the management of case reports involving sports-related dental trauma. Despite the frequent occurrence of dental injuries among athletes, the use of mouthguards is not widely promoted among either children or adults [32, 33]. Farhadiane et al. [34] demonstrated that children who were not aware of mouthguards were 5.44 times more likely to experience a dental injury than those who were aware of mouthguards, maxillofacial injuries are even more common among professionals than amateurs (86%, 42.1%, respectively) [17]. This aligns with the case reports included in this review, where no athlete used protection at the time of the trauma. The highest prevalence of sports-related dental trauma is observed in males [35, 36], which corroborates with the findings in this review. Among the eight studies evaluated, seven involved male patients, with an average age of 21 years, indicating that younger patients are more often affected. Regarding the type of trauma, most studies (four) involved fractures, and concerning the location, six cases involved the upper anterior region, consistent with the literature [35, 36]. Additionally, there was a high level of heterogeneity in the sports involved including basketball, soccer, golf, volleyball, and bicycling.

Emergency care plays a critical role in determining trauma outcomes as early intervention is associated with significantly better prognoses [37]. Karp et al. [27] showed that when the patient’s parents sought dental care immediately after the avulsion the established management was carried out and 865 days post-trauma the tooth exhibited normal mobility with no clinical or radiographic signs of replacement resorption or pathology. Conversely, Chikkanarasaiah et al. [28] describe another incisor avulsion case in which dental care was sought one and a half months after the trauma. The repositioning attempts were not possible and a biological removable functional space maintainer was needed. Given this, we can highlight the importance of the knowledge of coaches and parents in instituting first aid on site during instances of trauma. To address this need, the International Association of Dental Traumatology has written guidelines for managing traumatic dental injuries, providing a sequence of recommendations and instructions to be followed from the immediate aftermath of the trauma to arrival at dental care [37, 38]. Each type of trauma has specific protocols which coaches need to be knowledgeable to handle each situation appropriately. Overall, the case reports presented by select studies of this systematic review showed that there is no standard protocol of the type of management adopted or treatments with better prognoses. Treatment included endodontics, periradicular treatments, splinting, orthodontics, and the use of stabilizing plates. In instances where it was not possible to retain the tooth, dental implants [29] and temporary removable prostheses [28] were utilized. This variability is likely due to different professionals from various regions, use of complementary imaging exams, and symptoms of patients.

The choice of a proper management strategy for a sports-related dental trauma has to consider multiple interrelated factors such as socio-demographic characteristics, the patient’s oral health status, the type, intensity, and mechanism of trauma, and the specific dental region affected which may affect the diagnosis, treatment, and prognosis. Thus, clinical decisions must be individually tailored. The location of the affected teeth and the patient’s age are particularly important, as they significantly shape the clinical presentation, treatment options, and expected outcomes. Anterior teeth—particularly maxillary central incisors—are more frequently injured in sports-related trauma due to their exposed position and are often associated with esthetic concerns. These injuries typically require timely and conservative management focused on preserving function and appearance. Posterior teeth, while less commonly affected, may experience trauma related to occlusal forces or blunt impacts, often necessitating more complex restorative or surgical interventions. Management strategies also vary between pediatric and adult populations. In children, injuries may involve primary teeth or immature permanent teeth, requiring minimally invasive techniques and careful long-term follow-up to avoid disrupting dental development. In adults, with fully developed dentition, a wider range of restorative or endodontic treatments may be applicable. Recognizing and addressing these clinical nuances is essential for developing individualized treatment plans, enhancing patient outcomes, and guiding the creation of standardized protocols for managing sport-related dental trauma [39–41].

An important aspect to address is the utilization of complementary investigations and examinations that assist in the accurate diagnosis of dental trauma [42]. The use of imaging provides critical information for accurately diagnosing trauma, which is essential for determining the appropriate treatment [42, 43]. This systematic review found that most of the studies used periapical radiography as a complementary examination for diagnosis and follow-up, and some cases combining it with cone-beam CT and/or ultrasound exams. Thermal sensitivity tests were mentioned in only two of the reviewed reports. This simple yet important test provides information on pulp vitality, allowing for the assessment of whether sensitivity has been recovered or lost after immediate treatment [44, 45]. Importantly, advanced imaging techniques, such as cone-beam CT, have been shown to enhance diagnostic accuracy in traumatic dental injuries, particularly in detecting root fractures, alveolar bone involvement, and the extent of luxation injuries. Compared to conventional intraoral radiography, cone-beam CT provides three-dimensional visualization, which can significantly impact treatment planning and decision-making in complex cases [41]. However, despite its advantages, cone-beam CT is not yet universally adopted due to concerns regarding cost, radiation exposure, and accessibility [46]. Future studies should evaluate how cone-beam CT and other imaging modalities influence treatment success, long-term prognosis, and overall patient outcomes. Additionally, research comparing CBCT with conventional radiographic techniques in terms of cost-effectiveness and clinical applicability would be beneficial.

Follow-up care is a vital component of dental trauma management, facilitating the monitoring of healing, identifying complications, and adjusting treatment as needed [47, 48]. These visits provide opportunities to evaluate the effectiveness of initial treatment, adjust care plans, and implement additional therapeutic measures if necessary [47, 48]. Follow-up time is a subject of controversy in the literature, depending on the region and type of trauma. The International Association of Dental Traumatology has written guidelines that indicate the appropriate follow-up time for each situation [37, 38, 49]. Among the selected studies of this systematic review, two did not include follow-up visits [28, 31]. In the other studies, follow-ups ranged from a minimum of 7 months to a maximum of 13 years. This variation may be related to the professional decision in the face of the complexity and individuality of each case, as well as to the findings in each follow-up visit, which may include the patient’s response to treatment, the presence of complications, and the results of clinical and imaging exams. These factors necessitate a tailored approach to the duration and frequency of follow-up, ensuring that each patient receives the most appropriate and effective care based on their unique circumstances. Recognizing this heterogeneity is essential for appropriately interpreting study results and applying best practices in managing dental trauma, ensuring a personalized and effective approach for each patient. Therefore, consistent and thorough follow-up is indispensable in ensuring the best possible outcomes for patients who have experienced dental trauma [47, 48].

Standardized guidelines for the diagnosis, treatment, and follow-up of sport-related traumatic dental injuries are essential to improve consistency in clinical management while allowing for individualized treatment based on patient-specific factors and trauma mechanisms. Given the variability in injury patterns between contact and non-contact sports, diagnostic protocols should incorporate different imaging modalities, such as periapical radiographs and cone-beam computed tomography to enhance accuracy accordingly with the justification. Treatment strategies should be adaptable, considering factors like dentition stage, trauma severity, and time to intervention. Contact sports are more frequently associated with dental and bone fractures, often requiring stabilization and surgical intervention, whereas non-contact sports more commonly lead to avulsions, emphasizing the importance of immediate reimplantation and endodontic management [14]. Furthermore, standardized follow-up intervals and success criteria—including tooth survival, function preservation, and patient-reported outcomes—should be established to improve prognostic assessment [39].

Even after a successful outcome in a trauma case, prevention remains important to reduce injury incidence [50]. In cases of sports-related dental trauma, it is crucial to emphasize the use of mouthguard during sports activities. The case report by Stojanac et al. [24] demonstrated the successful maintenance of three dental elements following a horizontal fracture in the cervical third. However, seven months post-treatment, the patient sustained a new fracture during a basketball game, leading to the recommendation for tooth extraction. The American Dental Association (ADA) advocates for the use of mouthguards, particularly in high-contact sports [51]. However, despite this recommendation, compliance remains inconsistent across different sports and competition levels. Factors such as lack of awareness, discomfort, and perceived impact on performance contribute to low adherence rates [52]. Future research should focus on assessing the effectiveness of different mouthguard designs, promoting their mandatory use in sports regulations, and improving athlete education on their benefits.

One must bear in mind that among the eight studies included, 75% of the reported cases were associated with contact sports. When comparing case descriptions across different injury mechanisms in sports activities (contact vs. non-contact), distinct patterns in trauma occurrence were observed. Contact sports were more frequently associated with bone and/or dental fractures due to the higher likelihood of direct physical impact, while non-contact sports were more commonly linked to dental avulsion, often resulting from falls or accidental blows. Similarly to the general assessment, when analyzed separately the characteristic management of each injury mechanism, the approaches did not appear to be consistent, highlighting significant variability across all described treatment stages. This variability underscores the need for tailored preventive strategies, such as the widespread adoption of mouthguards in contact sports and improved first-aid awareness for dental avulsions in non-contact sports.

The clinical significance of this systematic review lies in its ability to compile and analyze various case reports related to sports-related dental trauma, providing crucial insights for clinical practice. By examining how different cases were managed, patterns and gaps in dental trauma management were identified, highlighting the need for effective preventive strategies and intervention. Additionally, the review underscores the importance of ongoing follow-up after trauma to optimize treatment outcomes and minimize long-term complications. These findings are essential for guiding oral health professionals, sports instructors, parents, and athletes in the prevention and appropriate management of dental trauma associated with sports activities.

The limitations of this systematic review include its exclusive focus on a small number of case reports and the heterogeneity in their reporting methods, which may affect the comparability and generalizability of the findings. Although all included studies demonstrated low risk of bias according to the JBI Critical Appraisal Checklist for Case Reports, the inherent nature of case reports—often susceptible to selection and reporting biases—must be acknowledged, as these factors can still influence the strength of the conclusions. Additionally, variability in the level of detail reported, particularly regarding trauma types, follow-up durations, and diagnostic methods, may have hindered a comprehensive evaluation of treatment outcomes. Despite these limitations, the review highlights a notable gap in the literature regarding the management of sport-related dental trauma. The synthesis of available data reinforces the urgent need for future studies with more robust methodological designs and standardized protocols for diagnosis, treatment, and follow-up. Such efforts are essential to enhance the consistency, reliability, and clinical applicability of findings in this field.

Overall, considering the synthesis of the included studies, the present systematic review highlights a significant lack of consistency in the clinical management of sport-related dental trauma, reinforcing the need for standardized protocols. An example of a potentially transferable approach can be found in the study by Palermi et al. (2022) [53], which assessed the feasibility and acceptability of the pGALS (pediatric Gait, Arms, Legs, and Spine) examination during pre-participation sports evaluations in children. Although pGALS is designed for musculoskeletal assessment, its methodological structure offers a valuable model for developing guidelines for dental trauma management in sports settings. Implementing a rapid and efficient dental screening protocol—capable of identifying pre-existing injuries or risk factors—during both pre- and post-participation evaluations could enhance early detection and improve outcomes. Such screening should be practical, easily administered by non-dental professionals, and acceptable to athletes and their guardians. In parallel, educating and training sports medicine professionals to recognize and manage dental injuries, especially in environments where dental specialists are not readily available, could significantly improve immediate care. Standardized triage and referral protocols would further support timely and effective interventions. Nevertheless, it should be recognized the potential barriers to implementing these measures across diverse sporting contexts. These challenges include limited access to dental professionals, discrepancies in available resources between amateur and professional levels, varying national guidelines or policies, and a general lack of awareness regarding the role of oral health in injury prevention and athletic performance. Addressing these issues will require a multidisciplinary effort involving institutional support, policy development, and targeted educational initiatives aimed at athletes, coaches, and healthcare providers.

## Conclusion

This systematic review highlights the significant heterogeneity in the management of sports-related dental trauma across different sports and competitive levels. Despite the existence of guidelines from the International Association of Dental Traumatology (IADT), their application varies due to differences in awareness, training, and accessibility of dental care in sports settings. To improve first aid management and long-term outcomes, it is crucial to enhance education of coaches and sports professionals, particularly regarding the importance of mouthguard use, in preventing dental injuries and promoting better clinical prognosis. Future research should focus on identifying the key barriers to guideline implementation, assessing the effectiveness of educational interventions, and developing standardized emergency protocols tailored to different sports. Additionally, further studies are needed to evaluate the long-term outcomes of various management strategies and the protective role of mouthguards and other preventive measures in reducing dental trauma incidence and severity.

## Supplementary Information

Supplementary Material 1.

## Data Availability

All of the material is owned by the authors and/or no permissions are required. The datasets analyzed during the current study are available in the submitted manuscript file. The datasets analyzed during the current study are available in the submitted manuscript file.
